# Characterization of Chemically Induced Ovarian Carcinomas in an Ethanol-Preferring Rat Model: Influence of Long-Term Melatonin Treatment

**DOI:** 10.1371/journal.pone.0081676

**Published:** 2013-12-18

**Authors:** Luiz Gustavo A. Chuffa, Beatriz A. Fioruci-Fontanelli, Leonardo O. Mendes, Wagner J. Fávaro, Patricia Fernanda F. Pinheiro, Marcelo Martinez, Francisco Eduardo Martinez

**Affiliations:** 1 Departamento de Anatomia, Instituto de Biociências, UNESP – Universidade Estadual Paulista, Botucatu-SP, Brazil; 2 Programa de Pós-Graduação em Biologia Celular e Estrutural, Instituto de Biologia, Universidade Estadual de Campinas – UNICAMP, Campinas-SP, Brazil; 3 Departamento de Anatomia, Biologia Celular e Fisiologia e Biofísica, UNICAMP – Universidade de Campinas, Campinas-SP, Brazil; 4 Departamento de Morfologia e Patologia, UFSCar – Universidade Federal de São Carlos, São Carlos-SP, Brazil; University of Quebec at Trois-Rivieres, Canada

## Abstract

Ovarian cancer is the fourth most common cause of cancer deaths among women, and chronic alcoholism may exert co-carcinogenic effects. Because melatonin (mel) has oncostatic properties, we aimed to investigate and characterize the chemical induction of ovarian tumors in a model of ethanol-preferring rats and to verify the influence of mel treatment on the overall features of these tumors. After rats were selected to receive ethanol (EtOH), they were surgically injected with 100 µg of *7,12*-*dimethyl-benz[a]anthracene* (DMBA) plus sesame oil directly under the left ovarian bursa. At 260 days old, half of the animals received i.p. injections of 200 µg mel/100 g b.w. for 60 days. Four experimental groups were established: Group C, rats bearing ovarian carcinomas (OC); Group C+EtOH, rats voluntarily consuming 10% (v/v) EtOH and bearing OC; Group C+M, rats bearing OC and receiving mel; and Group C+EtOH+M, rats with OC consuming EtOH and receiving mel. Estrous cycle and nutritional parameters were evaluated, and anatomopathological analyses of the ovarian tumors were conducted. The incidence of ovarian tumors was higher in EtOH drinking animals 120 days post-DMBA administration, and mel efficiently reduced the prevalence of some aggressive tumors. Although mel promoted high EtOH consumption, it was effective in synchronizing the estrous cycle and reducing ovarian tumor mass by 20%. While rats in the C group displayed cysts containing serous fluid, C+EtOH rats showed solid tumor masses. After mel treatment, the ovaries of these rats presented as soft and mobile tissues. EtOH consumption increased the incidence of serous papillary carcinomas and sarcomas but not clear cell carcinomas. In contrast, mel reduced the incidence of sarcomas, endometrioid carcinomas and cystic teratomas. Combination of DMBA with EtOH intake potentiated the incidence of OC with malignant histologic subtypes. We concluded that mel reduces ovarian masses and the incidence of adenocarcinomas in ethanol-deprived rats.

## Introduction

OC is the most lethal of all of the gynecological malignances [1]. The high incidence of OC has been attributed to primordial factors that are associated with ovarian senescence, such as oocyte depletion, low levels of steroid production, and increased levels of circulating gonadotropins [2]. OC has been presumed to arise from precursor lesions involving the ovarian surface epithelium (OSE) or ovarian epithelial inclusion cysts, which exhibit morphological features of the reproductive tract epithelium (or Mullerian aspects). Mullerian inclusion cysts may develop from the tubal epithelia by exfoliation or tubal-ovarian adhesions, during ovulation repair, or may even develop from endometrial cells via endometriosis [3]–[5]. Furthermore, several events associated with ovulation are hypothesized to be linked to OC initiation and progression, including proliferation of the OSE to repair ovulation-induced wounds on the ovarian surface [6], which may result in DNA damage; elevation of gonadotropin levels, stimulating the growth of the OSE [7]; and ovulation-induced inflammation, leading to the generation of reactive oxygen species (ROS) [8]. Approximately 90% of OCs are believed to arise from the OSE, while the remainder of ovarian neoplasms seem to be closely split between stromal and germ cell origins [9], [10]. The lack of an adequate screening method to detect the early stages of the disease and its progression to chemoresistance has prevented appreciable improvements in the survival rate of patients suffering from OC.

Unfortunately, a paucity of OSE tumor animal models exist that can be extrapolated to tumors occurring in women. 7,12-dimethylbenz[a]anthracene (DMBA) is a well-known polycyclic aromatic carcinogen that is capable of inducing the initiation, promotion and progression of tumors [11]. After an optimal period of ovarian instillation, the observed neoplasms develop into massive tumors and occasionally evolve to intraperitoneal nodules that are accompanied by bloody ascites [12], [13]. Importantly, the histologic types of experimental ovarian tumors may vary based on the animal strain used, the age of the animals, and the use of the carcinogen (the amount, route of administration, and method of induction). To date, the effective detection and specific treatment of OC remains a significant clinical challenge.

Evidence that suggests a plausible biological relationship exists, revealing that higher doses of alcohol consumption cause anovulation, and hence, protection against ovarian carcinogenesis [14]. Conversely, an American hospital-based study reported a direct association between alcohol drinking and OC risk, while other studies detected no relationship [15]–[17]. Despite these conflicting results, an animal model of OC in which the hormone profile and ovarian metabolism were severely disrupted by ethanol consumption would represent a good choice for investigating the potential of a carcinogen together with a risk factor (e.g., alcohol intake). Our ethanol-preferring rat strain, UChB, was derived from the original Wistar rat, and has been selectively bred at the University of Chile for decades [18]. These animals are considered to be a special model that can be used to understand alcoholism-linked characteristics, such as those observed in human diseases. A model possessing the histologic patterns appropriate to those found in human ovarian tumors is needed to evaluate new chemopreventive compounds.

Melatonin (*N*-acetyl-5-methoxytryptamine) is an indoleamine that is produced by the pineal gland and secreted in a circadian manner overnight [19]. Melatonin has indisputably been implicated as a therapeutic agent in several cases, including the treatment of reproductive cancers [20], [21]. Due to its functional versatility, melatonin has been recognized as having antioxidant, oncostatic and immunomodulatory properties [22]. Some cytokines (IL-2, -4 and -12) and factors (TNF-alpha and INF-gamma) displaying immunotherapeutic potential have been associated with melatonin and are under investigation for use as adjuvant therapies for cancer [23], [24]. Furthermore, multiple reports have indicated that melatonin prevents the initiation, promotion and progression of tumors [22], [23]. The efficacy of melatonin in limiting the proliferation of tumor cells has also been demonstrated to be dependent upon the timing of administration, with the optimal timing being the end of the most suitable light phase [25]. The oncostatic effects of melatonin have also been shown to be more pronounced in hormone-dependent tumors (e.g., breast and ovarian cancers) [20]. In an effort to better understand this issue, the present study was designed to characterize the chemical induction of ovarian tumors in a model of ethanol-preferring rats, and to investigate the role of melatonin in the overall features of ovarian tumors.

## Materials and Methods

### Animals and Experimental Design

Eighty adult (60 day old) UChB (a non-isogenic model of ethanol-preferring rats that was developed by selective breeding to study the effects of chronic ethanol consumption) female rats, weighing ±200–230 g, were obtained from the Department of Anatomy, Bioscience Institute/Campus of Botucatu, UNESP–Univ Estadual Paulista. The rats were individually housed in polypropylene cages containing laboratory-grade pine shavings as bedding and maintained under controlled room temperature (23±1°C) and lighting conditions (12 h light and 12 dark photoperiods, with the lights switched on at 6 a.m.). Filtered tap water and standard rodent chow (3074 SIF, Purina Ltda., Campinas, SP, Brazil) containing (by weight) 19.90% protein, 30.03% carbohydrate, 3.80% fat, 13.36% fiber, and 3.00 kcal/g of metabolizable energy were provided *ad libitum*. All animals were divided into two arms (n = 40/group): an EtOH group, in which the rats had access to a 10% (v/v) ethanol solution *ad libitum* (free choice of water or ethanol), and a control group, which was composed of ethanol-naïve rats without access to ethanol. When the UChB EtOH rats had reached 65 days of age, they were given a choice between two bottles containing either water (1) or a 10% (v/v) ethanol solution (2) *ad libitum* over a period of 15 days. After this period, animals displaying ethanol consumption higher than 2.0 g of EtOH/kg/day (ranging from 4 to 5 g of EtOH/kg/day) were selected according to the procedure outlined by Mardones and Segovia-Riquelme [18]. In the present study, the preference ratio associated with ethanol-seeking behavior was approximately 65%.

At 80 days of age, all of the animals were chemically induced via injection of DMBA (Sigma Chemical Co, St Louis, MO) directly into the left ovarian bursa (please see item 2.3 of the Methods section). Over the subsequent 180 days of the experimental period, the rats were followed, and tumor development was observed using ultrasonography. Ovarian size (volume and diameter) was used as a reference parameter during tumor development.

**Figure 1:**
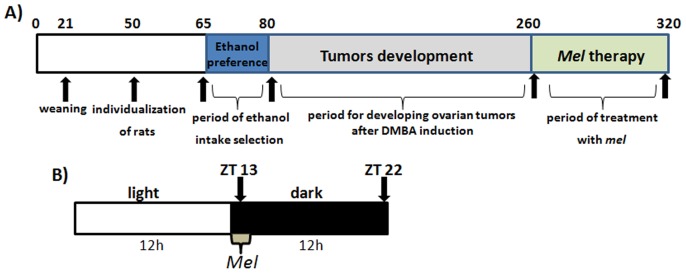
Detailed schedule of the experimental design. (**A**) Chronological scheme of ethanol intake, ovarian tumor induction and *mel* treatment (days). (**B**) Schematic protocol that was used for daily melatonin administration based on Zeitgeber Time (ZT), corresponding to environmental circadian time. ZT 13: Melatonin administration period. ZT 22: Period of euthanasia.

The animals designated to receive mel (n = 40) were administered i.p. doses of 200 µg/100 g of BW during the nocturnal period over the course of 60 consecutive days (please see item 2.4 of the Methods section and Fig. 1A).

For this proposal, the rats were finally divided into four groups: Group C, composed of DMBA-induced animals that did not consume ethanol; Group C+EtOH, composed of DMBA-induced animals that consumed 10% (v/v) ethanol during ovarian tumor development (OTD); Group C+M, composed of DMBA-induced animals that received melatonin as therapy; and Group C+EtOH+M, composed of DMBA-induced animals that consumed 10% (v/v) ethanol during OTD and received melatonin as therapy. After the experimental period, the females (whether cycling or not) were anesthetized and euthanized by decapitation (during the early morning at 4 a.m.) prior to further sample collection.

### Ethics Statement

To minimize pain, suffering or distress during experimentation, all of the animals were anesthetized with ketamine (50 mg/kg) and xylazine (10 mg/kg) prior to decapitation so that they were rendered unconscious. At 320 days of age, euthanasia was performed in accordance with the Canadian Council on Animal Experimentation. The present experimental protocol was previously accepted by the Ethical Committee of the Institute of Bioscience/UNESP (CEEA – Comitê de Ética em Experimentação Animal), Campus of Botucatu, SP, Brazil (Permit Number: 382).

### Ovarian Tumor Induction Procedure

After being selected to consume ethanol, all of the animals (n = 80) were anesthetized with 10% ketamine (60 mg/kg, i.p.) and 2% xylazine (5 mg/kg, i.p.) during the estrous stage. After the administration of anesthesia (1.2 mL/100 g of BW), the left flank region of the skin was cleaned with iodine and 70% ethanol. A 2 -cm incision was made through the skin and abdominal wall, and the ovaries were accessed by grasping the fat pad near the left kidney. After exteriorization, the left ovary was injected with a single dose of 100 µg of DMBA dissolved in 10 µL of sesame oil, which was used as the vehicle [26], directly under the ovarian bursa, and returned intact to the body cavity. The muscle layer and skin was closed using a 3-0 silk suture (Ethicon Inc., México, MX). Sham surgery was conducted on the right ovary using the vehicle only. Antibiotic (10^5^ units of benzylpenicillin potassium) was administered i.p. for the prophylactic treatment of infections before the abdominal wall was closed.

### Mel Administration Procedure

Mel (M-5250, Sigma-Aldrich Chemical Co., St. Louis, MO, USA) was dissolved in 0.04 mL of 95% ethanol and diluted in 0.3 mL of 0.9% NaCl (vehicle) at a steady-state concentration of 0.3 mg/mL. The mixture was then injected i.p. in a total volume of 3.4 mL/kg of BW. All of the treated and untreated animals were injected with a vehicle solution. For the melatonin treatments, i.p. injections at doses of 200 µg/100 g of BW were administered daily for 60 days [27] in the evening (between 18∶30–19∶00 hours, at ZT 13, as shown in Fig. 1B).

### Evaluation of Estrous Cycle and Nutritional Parameters

During the post-induction period, estrous cycles were monitored daily (at 9 a.m.) using a vaginal swab technique [28]. Fresh samples were transferred to clean slides and examined under an optical microscope. In addition, body weight gains, food consumption, and water/ethanol intake were measured every day at 9 a.m. for a period of each five days. After the melatonin treatment period, all of the rats were monitored by vaginal swabbing in a dark room using red dim illumination for subsequent euthanasia (anesthesia followed by decapitation) during the early morning at 4 a.m. (or Zeitgeber Time (ZT) 22, corresponding to the environmental circadian time, as shown in Fig. 1B).

### Determination of Plasma Mel Concentrations

After blood collection (4 a.m.), mel was extracted from the plasma (n = 10 samples/group) using HPLC grade methanol and was then separated on columns (Sep-Pak Vac C-18, reverse phase, 12.5 nm) (Water Corporation, Milford, Massachusetts, USA). Thereafter, 50 µL of reconstituted samples were assayed using a Coat-a-count Melatonin ELISA Kit and measured at 405 nm. The intra-assay coefficient of variation was 4%. All of the samples were assayed in duplicate at the same time to avoid inter-assay variability. All of the reagents and microtiter plates were provided by IBL (IBL International, Hamburg, Germany). The concentrations of melatonin are reported as pg/mL.

### Ovarian Histology Analyses

After euthanasia, all of the animals were necropsied and examined for abnormalities. Pathologically altered organs, female reproductive tracts, and organs/tissues related to body metabolism (liver, kidneys, pancreas, intestines, lymph nodes, and spleen) were removed during necropsy. In rats with evident ovarian tumors, specimens were also excised and frozen in liquid nitrogen, followed by storage at −80°C. For the histological analyses, the ovaries were fixed in 10% (v/v) buffered formalin (for 24 h), transferred to 70% ethanol, dehydrated, and paraffin embedded. Sections that were 5-µm thick were generated, and every 20^th^ section was stained with hematoxylin and eosin (H&E). Histopathological analyses were conducted by a pathologist with expertise in animal malignancies. The ovarian tumors were subtyped according to the histological aspects of the neoplasms.

### Statistical Analysis

The values are presented as the means ± standard deviation (SD). The data analyses were performed using two-way ANOVA (analysis of variance) for two independent factors (ethanol consumption and mel treatment). Significant results were subjected to *post-hoc* testing using Tukey’s test, and statistical significance was set at p<0.05. *GraphPad Instat Version 4* statistical software and *Sigma Plot Version 11.0* graphing software were used in this study.

## Results

### Nutritional Parameter and Estrous Cycle Examinations

During the experimental period, all of the animals were monitored and handled based on the same criteria. The amount of food intake and energy intake (derived from the feed and ethanol) did not differ between the experimental groups (p>0.05). However, the animals in Group C, independent of mel treatment, consumed more water than those in Group C+EtOH, regardless of treatment. Ethanol consumption was unaltered after ovarian tumor induction. When mel was administered, the amount of ethanol consumed increased after the first week through the end of the treatment period (34% increase in Group C+EtOH+M as compared to Group C+EtOH, as shown in Table 1).

**Table 1:** Food consumption (g/day), energy intake (Kcal/day), and relative water and ethanol consumption (mL/100 g/day) in all of the experimental groups.

Parameters	Vehicle		Vehicle+Melatonin	
	C+EtOH	C	C+EtOH+M	C+M
Food Consumption	15.31±0.46	14.80±0.65	16.08±0.66	14.89±0.34
Energy Intake(food+ethanol)	47.83±1.17	49.59±1.26	49.90±1.50	46.36±1.90
Relative WaterIntake	7.36±0.35	12.89±0.22^a^	8.78±0.76	16.88±0.44^bc^
Relative EthanolIntake (PT)	4.86±0.37	0.00	5.28±0.12	0.00
Relative EthanolIntake (AT)	5.16±0.89	0.00	7.88±0.62^a^ *	0.00

The values are expressed as the means ± SD. N = 20/group.

^a, b, c^ p<0.05 *versus* Groups C+EtOH, C and C+EtOH+M, respectively. Two-way ANOVA followed by Tukey’s test. PT: Prior to melatonin treatment. AT: After melatonin treatment.

p<0.01 differs significantly from relative ethanol intake (PT) compared to Group C+EtOH+M.

After tumor induction, the estrous cycles of rats in both Groups C and C+EtOH were longer (25% and 30.3% increase compared to Groups C+M and C+EtOH+M, respectively), and displayed transitional phase irregularities, particularly prolongation in the diestrus phase (50% of the analyzed period). Notably, mel was effective in reducing both the duration of the estrous phase and the extension of the diestrus phase. The disturbances in the estrous phase did not differ statistically between the groups (Table 2).

**Table 2:** Evaluation of estrous cycle (days) and frequency (%) of persistent arrest in the estrous and/or diestrus stage (N = 20/group).

Groups	Duration of Cycles (days)	Persistent Estrous/Cycle (%)	Persistent Diestrus/Cycle (%)
C+EtOH	9.95±0.60	12.67 (0.3;26.67)	47.66 (3.1;65.00)
C	10.55±0.45	13.23 (0.9;30.00)	49.80 (4.4;58.97)
C+EtOH+M	7.45±0.44*	15.67 (2.5;24.67)	39.00 (8.56;29.33)^a^
C+M	7.35±1.10**	16.67 (3.1;23.00)	34.66 (6.33;46.67)^bc^

Values are expressed as the means ± SD and medians (minimum/maximum values).

p<0.01 and **p<0.05 differs significantly from Groups C+EtOH and C, respectively. Two-way ANOVA followed by Tukey’s test.

^a, b, c^ p<0.05 *versus* Groups C+EtOH, C and C+EtOH+M, respectively, as analyzed using the Kruskal-Wallis test followed by a *post-hoc* Dunn’s test.

### Body and Reproductive Organs Weights

No differences in the final body weights or body weight gains (p>0.05) were observed during the experimental period. The absolute and relative weights and sizes of the left ovaries were significantly higher in the animals in Groups C and C+EtOH, and treatment with mel reduced these ovarian masses by approximately 20% (Table 3). Only animals that had undergone left tumor induction and consumed ethanol displayed decreased right ovarian weights, and mel therapy promoted an additive effect in the presence of ethanol, restoring these weights to normal. Finally, the oviduct and uterine weights remained unchanged during the treatments (Table 3).

**Table 3:** Final body weights (g), body weight gains (%), ovarian sizes (mm), and absolute (g) and relative (g/100 g B.W) reproductive tissue weights in the four experimental groups.

Parameters	Groups			
	C+EtOH	C	C+EtOH+M	C+M
Final Body Weight	255±4.36	252±3.59	258±3.73	250±4.10
Body Weight Gain	28±0.4	26±0.6	29±0.4	25±0.8
Left Ovarian Weight*	0.24±0.04	0.22±0.01	0.19±0.02^a^	0.17±0.02^b^
Relative Weight of the Left Ovary*	0.09±0.01	0.09±0.01	0.07±0.01^a^	0.06±0.01^b^
Left Ovarian Size*	15.9±3.7	20.0±4.2	12.8±2.5^a^	16.4±3.7^b^
Right Ovarian Weight	0.048±0.00	0.056±0.00^a^	0.059±0.01^a^	0.051±0.01^c^
Relative Weight of the Right Ovary	0.02±0.00	0.02±0.00	0.03±0.00	0.02±0.00
Oviduct Weight	0.031±0.02	0.033±0.03	0.029±0.01	0.030±0.01
Relative Weight of the Oviducts	0.012±0.01	0.013±0.01	0.012±0.03	0.013±0.01
Uterine Weight	0.50±0.16	0.53±0.13	0.48±0.15	0.52±0.11
Relative Weight of the Uterus	0.15±0.04	0.16±0.06	0.18±0.09	0.18±0.08

The values are expressed as the means ± SD. N = 20/group.

^a, b, c^ p<0.05 *versus* Groups C+EtOH, C and C+EtOH+M, respectively. (*) indicates that the left ovary was chemically induced with DMBA. Two-way ANOVA followed by Tukey’s test.

### Incidence of Ovarian Tumors and Plasma Melatonin Levels

**Figure 2:**
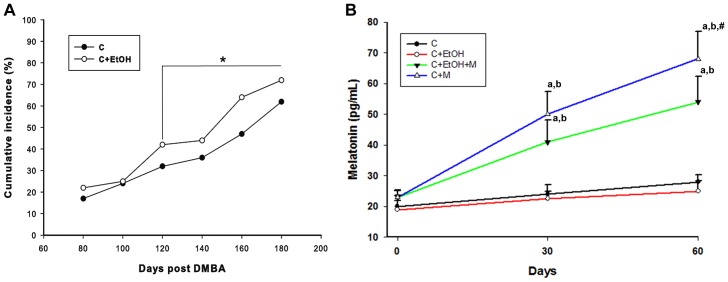
Period of tumor development and plasma melatonin levels during the treatment. Cumulative incidence (%) of ovarian tumors from 80 to 180 days post-DMBA injection (A). From 120 to 180 days post-DMBA induction, the ovaries of the animals in Group C+EtOH showed a significant incidence of tumor development. *P<0.05 vs. Group C. (B) Detection of plasma melatonin levels prior to treatment initiation (day 0) and after 30- and 60- days of treatment. ^a, b^ *P*<0.05 vs. Groups C and C+EtOH, respectively. ^#^ *P*<0.05 vs. 30 days of treatment in the same group.

The incidence of DMBA-induced ovarian tumors (the percentage of animals in each group that developed tumors) was higher in animals that consumed ethanol (Fig. 2A). Thus, at 80 days post-induction (DPI), the incidence was 22% in Group C+EtOH and 17% in Group C. At 100 DPI, the rate was 25% in Group C+EtOH and 24% in Group C. At 120 DPI, the rate was 42% in Group C+EtOH and 32% in Group C. At 140 DPI, the rate was 44% in Group C+EtOH and 36% in Group C. At 160 DPI, the rate was 64% in Group C+EtOH and 47% in Group C. At 180 DPI, the rate was 74% in Group C+EtOH and 60% in Group C (Fig. 2A). After 30 and 60 days of mel administration, both Groups C+M and C+EtOH+M displayed significant increases in plasma mel concentrations compared to Groups C and C+EtOH. After 60 days of treatment, only the rats in the C+M group showed elevations in mel levels compared to 30 days of treatment (Fig. 2B).

### Anatomopathological Ovarian Tumor Analyses

**Figure 3:**
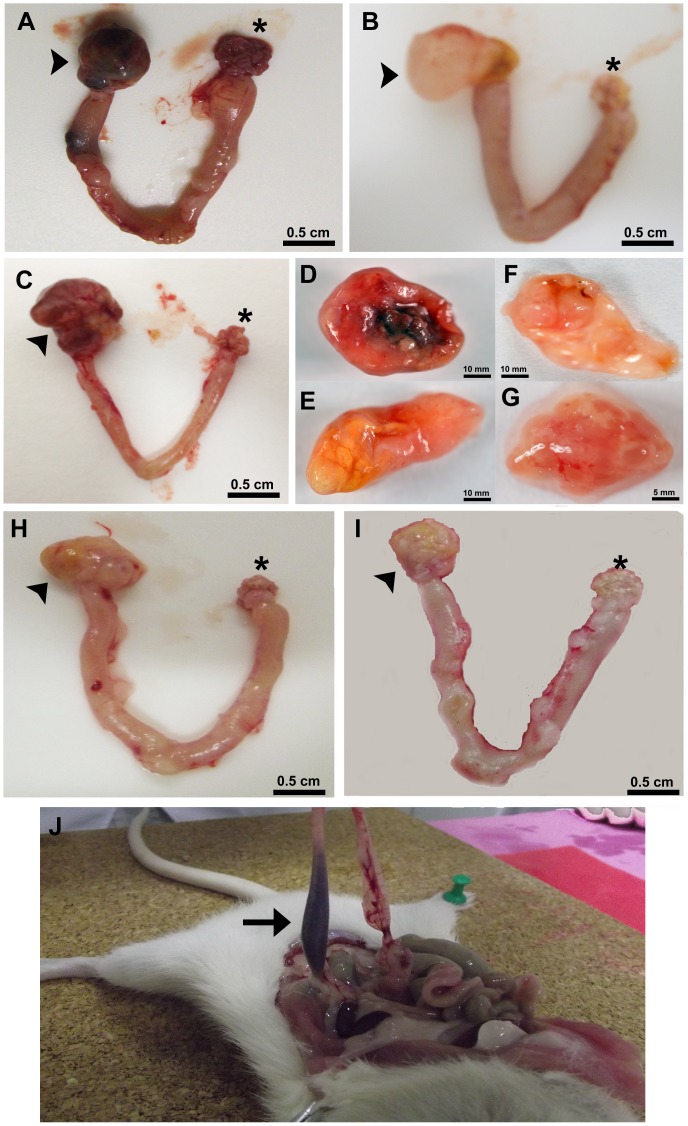
Photographs of the most common ovarian tumors 180 days post-DMBA induction. (A and C) The left ovary (arrowhead) of an animal from the C+EtOH group showing a solid mass with scattered necrosis. (B) The left ovary (arrowhead) of an animal from Group C, displaying a larger sized cyst containing serous secretions. High magnification of ovarian neoplasms in the C+EtOH (D), C+M (E), and C+EtOH+M groups (F), and a sham-operated right ovary (G). The left ovaries (arrowhead) of animals from the C+EtOH+M (H) and C+M (I) groups. (J) Left uterine horn (black arrow), displaying internal blood stasis. (*) indicates the intact right ovary (which was only treated with the vehicle). Proliferating cancer cells increased the ovarian masses to diameters >4-fold in size (left ovaries).

After sequential tumor development, the abdominal-pelvic cavity was opened to assess ovarian tumors. The left ovaries of rats from the C+EtOH group displayed evident solid masses with scattered necrotic spots and with no cysts (Figs. 3A and C). Furthermore, these animals exhibited blood stasis in the left uterine horn (Figs. 3J). The left ovaries of animals in Group C contained serous fluid-filled cysts (as shown in Fig. 3B; histopathological analyses of the tissue confirmed the presence of cystadenocarcinomas). Although the ovaries of the rats in the C+M and C+EtOH+M groups were increased in size (16.4±3.7 *vs.* 12.8±2.5 mm), great differences in the morphological constitution of these ovaries were observed, which were evidenced by soft and mobile tissues containing no adhesions (Figs. 3 E, F, H and I). The sham-operated ovaries (*) appeared to be intact with no macroscopic lesion.

### Histological Features of Ovarian Tumors

**Figure 4:**
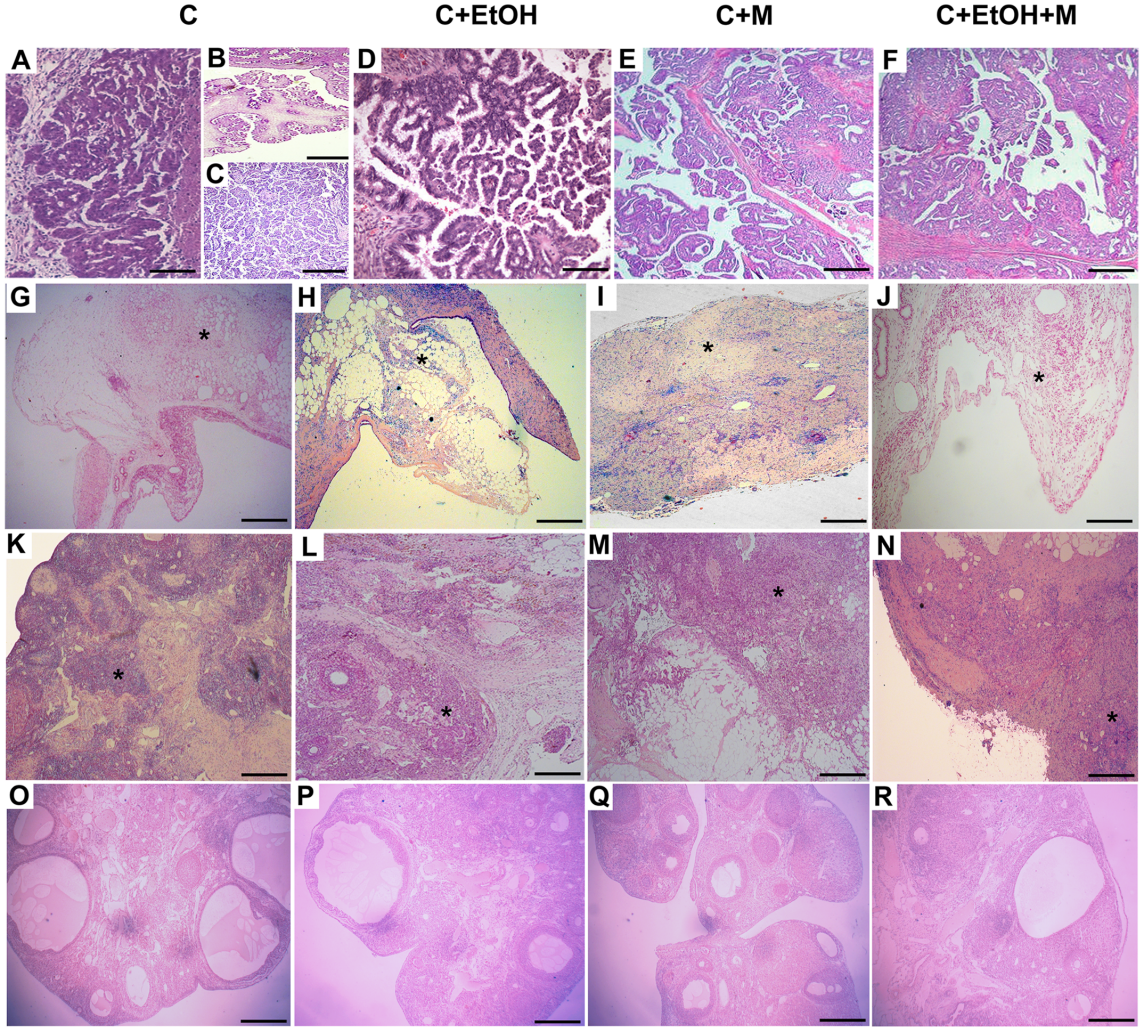
Photomicrographs of the three most prevalent intraovarian DMBA-induced ovarian tumors in UChB rats. In Group C, serous borderline tumors displayed an exophytic papillary architecture (**A and B**) mixed with extensive micropapillae formation, or tufts back-to-back (**C**). The majority of these adenocarcinomas exhibited invasive gland-like neoplastic structures invading the cortex. The C+EtOH (**D**), C+M (**E**) and C+EtOH+M (**F**) groups presented very complex micropapillary patterns, composed of columnar atypical cells, with no stromal invasion. Clear cell carcinomas (asterisk) with solid growths surrounding the stromal tissues were observed in Groups C (**G**), C+EtOH (**H**), C+M (**I**) and C+EtOH+M (**J**). Sarcoma cells (asterisk) were arranged in bundles and streams with spindle shapes and occupied extramedullary sites in Groups C (**K**), C+EtOH (**L**), C+M (**M**) and C+EtOH+M (**N**). Sham-operated right ovaries with developing and mature follicles and new corpora lutea in Groups C (**O**), C+EtOH (**P**), C+M (**Q**) and C+EtOH+M (**R**). Original low magnification, bar = 200 µm; H&E stain.

**Figure 5:**
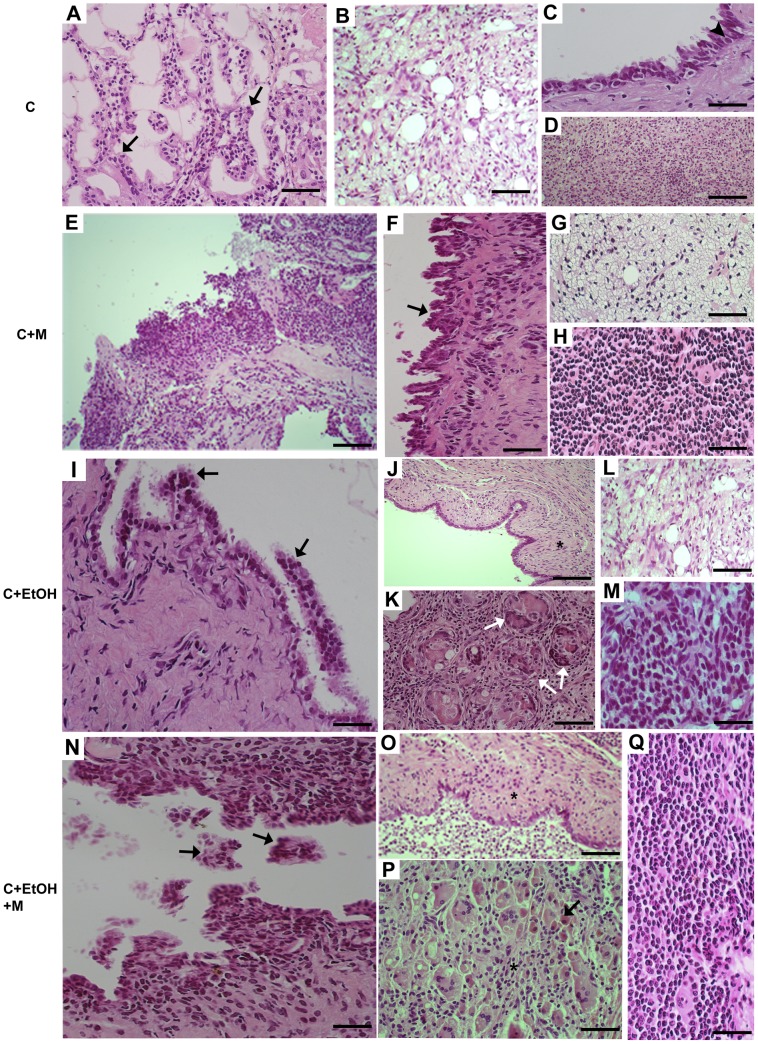
Histologic subtypes of ovarian tumors in the four experimental groups. In Group C: **A)** Serous carcinoma characterized by papillae formation with cellular budding (arrows), bar = 20 µm. **B)** Clear cell carcinoma composed of numerous vacuolated cells, bar = 20 µm. **C)** Ovarian carcinoma *in situ.* The epithelium is composed of stratified cells with loss of nuclear polarity and nuclear pleomorphism with chromatin clumping (arrowhead), bar = 20 µm. **D)** Sarcoma composed of non-epithelial malignant cells, bar = 20 µm. In Group C+M: **E)** Serous carcinoma with a pattern of papillae, bar = 50 µm. **F)** Serous papillary cystadenoma (borderline tumor) showing a higher complexity of cellular stratification, bar = 20 µm. **G)** Pattern of clear cell carcinoma with hobnail cells, bar = 20 µm. **H)** Sarcoma, bar = 20 µm. In Group C+EtOH: **I)** Serous carcinoma showing a section of a papillae (arrow), bar = 20 µm. **J)** Squamous carcinoma composed of squamous epithelial cells (asterisk), bar = 50 µm. **K)** Mature cystic teratoma with malignant content were scattered in the ovarian stroma (white arrows), bar = 50 µm. **L)** Clear cell carcinoma, bar = 20 µm. **M)** Sarcoma, bar = 20 µm. In Group C+EtOH+M, serous carcinoma showing a cross-section of a papillae (**N**), bar = 20 µm. **O)** Squamous carcinoma, bar = 50 µm. **P)** Ovarian carcinoma with the presence of specifically undifferentiated cells (asterisk). The stroma displayed a marked infiltration of inflammatory cells (arrow), bar = 20 µm. **Q)** Sarcoma, bar = 20 µm; H&E stain.

The majority of ovarian tumors were adenocarcinomas. After 180 days of ovarian tumor induction, the experimental groups displayed variations in the histotypes and grades of the tumors. Serous papillary carcinomas were more prevalent in animals from the C+EtOH (24/80) and C+EtOH+M (22/70) groups than in the C (10/60) and C+M (6/50) groups. Conversely, clear cell carcinomas were predominant in animals from the C (5/60) and C+M (4/50) groups compared to the animals in the C+EtOH (4/80) and C+EtOH+M (4/70) groups. Sarcomas were more prevalent in animals from the C (3/60) and C+EtOH (5/80) groups, and mel therapy was thought to have reduced their appearance (2/50 in Group C+M and 2/70 in Group C+EtOH+M). There was a low prevalence of undifferentiated carcinomas in Groups C (1/60), C+EtOH (1/80) and C+EtOH+M (1/70), and undifferentiated carcinomas were absent in Group C+M. Squamous cell carcinomas were present only in the groups that consumed ethanol, whether they had received mel (2/80 in Group C+EtOH and 2/70 in Group C+EtOH+M). Only Groups C (1/60) and C+EtOH (2/80) developed endometrioid carcinomas and mature cystic teratomas, respectively. Benign ovarian tumors were identified in Groups C (3/60) and C+M (5/50), and were more prevalent in the latter group (Table 4). No tumors with histological characteristics resembling either granulosa cell or germ cell tumors were observed. The most different types of ovarian carcinomas that were common in the four experimental groups are illustrated in Figures 4 and 5.

**Table 4:** Prevalence (%) of the different ovarian tumor subtypes, observed 180 days after DMBA injection in the four experimental groups.

Histologic Types	Groups			
	C	C+M	C+EtOH	C+EtOH+M
Serous Papillary	16.6%	12.05%	30.00%	31.33%
Clear CellCarcinoma	8.50%	7.63%	5.11%	5.95%
Sarcoma	5.00%	3.84%	6.25%	2.85%
UndifferentiatedCarcinoma	1.65%	–	1.25%	1.43%
Squamous CellCarcinoma	–	–	2.50%	2.86%
EndometrioidCarcinoma	1.66%	–	–	–
Mature CysticTeratoma	–	–	2.50%	–
Benign OvarianTumor	5.02%	10.07%	–	–

N = 20/group.

## Discussion

The present study is the first to demonstrate an association between DMBA and 10% (v/v) ethanol intake during ovarian tumor development, as well as the long-term effects of *mel* treatment on the general aspects of the tumors. The evidence supporting the effects of ethanol on DMBA-induced tumors in a classic model of UChB rats include the observations that: (a) ethanol increased the cumulative incidence of ovarian tumors (post-DMBA injection), (b) the weights and sizes of the tumors were increased after ethanol consumption, and (c) more tumors had malignant characteristics in the ethanol consumption group. Many studies have indicated that ethanol acts as a co-carcinogenic agent in several diseases [29]–[31]. Epidemiological studies reported that a direct correlation between ethanol consumption and the increased risk for developing ovarian cancer is dependent upon the amount and exposure time (chronic or more acute exposure), and that the effects of ethanol on ovarian tumorigenesis seem to be caused primarily by hormonal disturbances [15], [32]. We have already mentioned the most deleterious effects of chronic ethanol intake on the ovaries, such as alterations in estrogen- and progesterone-receptors (ER-alpha, ER-beta, PRA and PRB), increases in estradiol levels, high ROS production, and changes in the ovarian structure and ultra-structure [27], [33], [34]. Furthermore, ethanol and its metabolite, acetaldehyde, are capable of inducing DNA adduct formation and cytochrome P450-mediated ROS generation, while also depleting folate levels [29], [35], [36]. Because these metabolic alterations are linked to the risk of developing tumors, ethanol-preferring rats can be an excellent option for enhancing tumor development in the presence of an active carcinogen (e.g., DMBA).

In contrast, we report that mel treatment significantly improved the general aspects of ovarian tumors. Although mel administration produces alcohol cravings, but not food-seeking behavior, it can reduce both relative and absolute ovarian tumor weights and sizes without affecting body weight. We know that OC risk is strongly linked to an increased number of lifetime ovulations. Thus, a blockage of incessant ovulation using oral contraceptives, or even pregnancy, may protect the ovaries from OC [37]. In the present study, DMBA administration alone, or in combination with EtOH, induced a late prolonged estrous cycle in rats when the ovaries already displayed evidence of tissue lesions. However, no anovulatory cycles were observed. Conversely, mel restored estrous cycle duration to near normal, and also reduced the diestrus stage. The low water intake observed in UChB rats was due to increases in the ethanol/ethanol+water ratios, and not to mel treatment [27]. In support of these data, mel is involved in the acquisition of ethanol preference rather than the maintenance of ethanol intake, thereby providing evidence that melatonin antagonists may reduce the reinforcement of ethanol consumption [38]. Corroborating the ovarian tumor reduction data, Srinivasan et al. [39] reported that melatonin is effective in suppressing neoplastic growth in a variety of reproductive tract tumors, including ovarian tumors. The main mechanism seems to be due to the immunoenhancing properties of melatonin in stimulating T-helper cell responses by releasing interleukin-2 and -10 and interferon-γ. In addition, melatonin prevents free radical damage in normal and tumor tissues, and induces the apoptotic mitochondrial pathway by reducing Bcl2 expression and caspase-3 activity [40], [41]. There may also be an anti-proliferative effect of melatonin in hormone-dependent tumors in UChB rats, such as that evidenced in mice [42]. Under normal conditions, melatonin at doses of 200 µg/100 g of BW/day induced longer estrous periods and reductions in ovarian weight [43]. Our previous reports suggested that long-term mel treatment satisfactorily reduces ovarian lipoperoxidation and plasma estradiol levels, while enhancing tissue antioxidant defenses [27], [28]. Moreover, mel treatment was also associated with lower ovarian weights and estrous cycle synchronization [44].

In the present study, we determined the ovarian tumor subtype based on histological observations. Spontaneous development of epithelial cancers is rarer in rodent ovaries [45] than in the ovaries of human beings. One plausible explanation for the low incidence is the fact that the rat ovaries are surrounded by a membranous pouch, which provides natural protection against the local effects of carcinogens [46]. To date, most of the studies of DMBA-induced tumors in rodent species (rat or mice) have generated reproducible ovarian neoplasms using various simulation methods and routes of carcinogen administration [11], [26], [47], [48], including gavage, coated suture or local instillation, dosage, time of drug delivery, and appropriate time for tumor development and growth. Interestingly, 120 days post-DMBA induction, the incidence of ovarian tumors was significantly increased after ethanol consumption (60% in the controls *vs.* 74% in the EtOH-treated group), of which 48% were adenocarcinomas of surface epithelial origin. Therefore, the development of neoplasms in DMBA-treated rats that had undergone ovarian failure (constant ethanol exposure) demonstrates an elevated susceptibility to develop ovarian tumors. However, the exact mechanism(s) involved in this ovarian sensitivity require further investigation. Although ethanol has the ability to act systematically on the whole ovary, all of the ovarian tumors were originated from the surface epithelium or cysts. It is believed that tumor origin may be related more to disruptions of the OSE than to perturbations of follicular or stromal tissues. Some of the DMBA-induced lesions, especially those related to the surface epithelium, were similar to the isolated papillae or diffuse papillomatosis found in human OC. Additionally, other OSE-derived structures that are commonly described in humans (i.e., simple microcysts or serous cysts) were eventually observed in the rat ovaries. In the present study, the development of the putative precursor lesions associated with DMBA exhibited degrees of differentiation and progression (i.e., cellular atypia and dysplasia) varying from low- to high-grade malignant potential. Briefly, clear cell carcinomas and sarcomas displayed similar features to those that occur in women. Several studies have shown a stronger relationship between DMBA-induced OC and human OC, both at the morphological and molecular levels [48]–[50]. Although DMBA is not a known environmental carcinogen that is linked to OC, it may share mutagenic mechanisms with other polycyclic aromatic compounds that are present at high concentrations in air pollutants or in tobacco, both of which have been implicated to play roles in human cancer development.

Further strengthening our findings, plasma mel concentrations were significantly elevated in the animals from both the C+M and C+EtOH+M groups, which ensured that an effective long-term therapy schedule had been followed. Although ethanol intake had been described to inhibit plasma mel [51], it was not sufficient to alter the continuous availability of mel in the early morning during the treatment period. Additionally, the nocturnal peaks of mel appeared to exacerbate both locomotor activity and EtOH-seeking behavior, while no apparent influence on the daytime behavior of the animals was observed. A notable activity rhythm after the administration of exogenous mel is typically found in nocturnal species (e.g., rodents) [52].

In regard to advanced ovarian tumors, melatonin alone reduced the incidence of serous papillary carcinomas, while the combination of DMBA with ethanol increased their appearance, regardless of mel treatment. This may be attributed to the effects of ethanol more than to the effects of DMBA itself. Conversely, a low incidence of clear cell carcinomas was observed after DMBA plus ethanol intake, and mel did not seem to affect this tumor subtype. In both ethanol-consuming and control animals, a low incidence of sarcomas was observed following melatonin treatment. Undifferentiated carcinomas had a very low incidence, and mel treatment alone suppressed its development. Intriguingly, only the combination of DMBA and ethanol intake led to the development of squamous cell carcinomas, and mel did not affect these tumors. There was also a low incidence of endometrioid carcinomas and mature cystic teratomas in the DMBA- and DMBA+ethanol treated groups, respectively. A high incidence of benign ovarian tumors was observed in DMBA-treated rats, and mel treatment further increased the incidence. In this group, mel may have exerted a beneficial effect, as evidenced by the reduced incidence of malignant ovarian tumors.

Notably, no inhibitory effects of melatonin (1 and 100 nM) on the growth of ovarian cancer cells in culture medium enriched with 1 nM 17β-estradiol were observed in *in vitro* studies [53]. Taking into account the fact that various experimental conditions were modified, such as differing melatonin incubation times (from 48 to 144 h) with stimulatory repetition every 24 h, or the use of different culture media, the effects of melatonin may be undetectable due to these variations. In addition, *in vitro* studies using different doses of mel (50, 100 and 200 nM) are being conducted on primary OC cultures originating from UChB rats (preliminary data not shown).

Altering the progression of DMBA-initiated cells to the formation of a tumor is an incessant paradigm in the chemoprevention area. Studies conducted by our research group examining chemoprevention and chemoresistance to drugs using this rat model are currently underway to assess the effectiveness of melatonin in stimulating immune function and inhibiting ovarian tumorigenesis.

In summary, chronic ethanol consumption enhanced the incidence of ovarian carcinomas in the presence of intraovarian DMBA. Furthermore, the association of DMBA with ethanol intake caused an increase in ovarian tumor mass with a high prevalence of malignant histologic subtypes. Although mel treatment promoted significant increases in ethanol consumption, it was able to effectively reduce ovarian mass. In addition to these benefits, mel also reduced the incidence of ovarian adenocarcinomas in ethanol-deprived rats. These data represent an important benchmark for understanding ovarian cancer development in a model of ethanol-preferring rats, and also suggest that melatonin may potentially be used as adjuvant therapy.
